# Exploring the interplay of clinical, ethical and societal dynamics: two decades of Medical Assistance in Dying (MAID) on psychiatric grounds in the Netherlands and Belgium

**DOI:** 10.3389/fpsyt.2024.1463813

**Published:** 2024-09-11

**Authors:** Monica Verhofstadt, Radboud Marijnissen, Daan Creemers, Sanne Rasing, Lizanne Schweren, Sigrid Sterckx, Koen Titeca, Sisco van Veen, Rosalie Pronk

**Affiliations:** ^1^ End-of-life Care Research Group, Ghent University, Ghent, Belgium; ^2^ University of Groningen, University Medical Center Groningen (UMCG), Department of Psychiatry, Groningen, Netherlands; ^3^ Geestelijke Gezondheidszorg (GGZ), Oost Brabant, Oss, Netherlands; ^4^ Behavioural Science Institute, Radboud University, Nijmegen, Netherlands; ^5^ 113 Suicide Prevention, Amsterdam, Netherlands; ^6^ Bioethics Institute Ghent, Ghent University, Ghent, Belgium; ^7^ Department of Psychiatry, General Hospital Groeninge, Courtrai, Belgium; ^8^ IQ Healthcare, Radboud University Medical Center (UMC), Nijmegen, Netherlands

**Keywords:** euthanasia, psychiatry, assisted suicide, mental illness, end-of-life care, Medical Aid in Dying

## Abstract

This paper explores recently emerging challenges in Medical Assistance in Dying on Psychiatric Grounds (MAID-PG), focusing on ethical, clinical, and societal perspectives. Two themes are explored. First, the growing number of young MAID-PG requestors and the public platform given to MAID-PG requests. Ethically, media portrayal, particularly of young patients’ testimonials, requires scrutiny for oversimplification, acknowledging the potential for a Werther effect alongside the absence of a Papageno effect. This highlights the need for better communication policies for media purposes. Second, cautionary considerations regarding psychiatric care adequacy are addressed. In MAID-PG this includes reasons underlying psychiatrist reluctance to engage in MAID-PG trajectories, leading to growing waiting lists at end-of-life-care centers. Addressing current shortages in psychiatric care adequacy is crucial, necessitating less narrow focus on short-term care trajectories and recovery beside transdiagnostic treatment approaches, expanded palliative care strategies, and integrated MAID-PG care.

## Introduction

The Netherlands and Belgium have led the way in establishing legal frameworks for Medical Assistance in Dying (MAID), with the uniqueness of not excluding MAID requests on psychiatric grounds (MAID-PG) per definition. For a summary of the legal criteria applicable in both countries, see **Box 1**.

Box 1 **Comparison between Belgium and the Netherlands of the legal due care requirements for MAID on psychiatric grounds.**

**Table:** 

Legal criteria	Belgium	the Netherlands
**MoDALITies^1^**	Euthanasia	Euthanasia Physician-Assisted Suicide
**Patient**	Mental Competence Adult or emancipated minor	12 years and older For ages 12 – 16: Parents/Guardians’ agreement For ages 16 – 18, Parents/Guardians must be involved in decision-making
**MAID Request**	Voluntary Well-Considered Repeated No external pressure	Voluntary Well-Considered
**Medical Condition**	No prospect of medical improvement No reasonable alternative solution	No reasonable alternative solution
**Suffering**	Persistent Unbearable Physical or psychological Irremediable Resulting from a serious and incurable condition caused by accident or illness	Irremediable Unbearable
**Physician's Duty to inform the Patient**	Patient’s Health condition Patient’s Life expectancy" MAID Remaining therapeutic options and their consequences Remaining palliative options and their consequences Physician’s Refusal and objections	Patient’s situation Patient’s prospects
**Independent Formal Advice on the MAID Request**	2 physicians, including 1 psychiatrist	1 Physician^2^
**Written Request**	Yes Revocable If the patient withdraws their MAID request and requests its removal from the medical record, it must be returned to the patient after removal	No
**Waiting Period**	At least 1 month between written request and implementation	No Waiting Period
**Performance**	Not included in the Law	Completed carefully
**Report and controle**	Federal Control and Evaluation Committee	Regional Review Committee
**Conscience Clause for Physicians**	Yes	Not included in the Law
**Duty to Refer**	Yes when not willing to participate in MAID or upon upon the patient’s request	Not included in the Law

^1^MAID encompasses two primary modalities: euthanasia, where a physician actively terminates a patient's life upon the latter’s explicit request, and physician-assisted suicide, where a lethal dose of medication is prescribed for self-administration. While the Belgian law explicitly mentions only the concept of "euthanasia" and lacks specific guidelines on its implementation, this has led to varying perspectives on whether 'physician-assisted suicide' is also covered by the Euthanasia Law. Efforts are currently underway to incorporate physician-assisted suicide into amendments of Belgian Euthanasia Law.

^2^According to the Dutch Guideline and the Euthanasia Code, at least 1 psychiatrist should be involved in MAID procedures on psychiatric grounds.

To date, Luxembourg and Spain also have legal frameworks that permit MAID-PG requests under specific conditions. Additionally, MAID-PG is legally ‘condoned’ in Switzerland. Finally, Canada is currently in the process of expanding its legislation on medical assistance in dying to include this specific patient group.

This paper focuses on the MAID-PG situation in the Netherlands and Belgium, and specifically on MAID-PG requests, which center on psychiatric illnesses as the primary grounds, excluding dementia. The primary underlying mental health conditions in MAID-PG cases often include multiple persistent psychiatric disorders, such as major depressive disorders, personality disorders, and autism spectrum disorder (1–4). Since the legalization of MAID in 2002 until 2023, Belgium reported 457 cases of MAID-PG (2, 5, 6), while the Netherlands reported 900 cases, constituting less than 1.5% of total MAID cases (7, 8). However, it is widely believed that the actual number of MAID-PG requests significantly exceeds these figures (2, 9, 10). Conversely, the proportion of physician-rejected and/or patient-withdrawn MAID requests is notably higher in psychiatric contexts compared to somatic medicine (2, 9, 11).

MAID-PG remains subject of ongoing ethical and clinical debates. This paper explores two under-discussed themes (‘key trends’). Firstly, the prominence of MAID-PG testimonials in the media, particularly those from young patients, who constitute the fastest-growing group of MAID-PG requestors (10, 12). Secondly, it highlights cautionary considerations regarding psychiatric care adequacy and the influence of societal factors. These encompass challenges such as a shortage of knowledge and expertise, as well as persistent reluctance of psychiatrists to engage in MAID-PG trajectories (1, 2, 9, 11, 13). These factors often result in the management of MAID-PG trajectories outside regular psychiatric facilities and growing waiting lists at MAID expertise centers. Additionally, it addresses broader issues in psychiatry, including accessibility concerns, the trend towards downsizing long-term care and fragmentation of care (14, 15), complexities in psychiatric care and suicide prevention (2, 16), the status of palliative care in psychiatry (17, 18), and how these issues may relate to MAID-PG.

Hence, the objective of this paper is to map and critically reflect on the abovementioned selected challenges within the MAID-PG debate, with a focus on ethical, clinical, and societal perspectives. We will start by assessing the role of prevailing public discourse in a broader context and its specific implications within psychiatry. By concentrating on these selected challenges, the paper aims to foster more informed and nuanced discussions about the societal impact, consequences, opportunities, and complexities associated with MAID-PG.

## Methods

In conducting this critical reflection, the authors leveraged their diverse backgrounds as researchers, clinicians, ethicists, and/or individuals with lived experience in psychiatry or MAID. This multifaceted approach facilitated a comprehensive exploration of the subject matter. The authors engaged in ongoing discussions via email exchanges and during three monthly meetings to collectively address their thoughts and concerns. These interactions provided a platform for iterative refinement of the paper, with each contributor offering insights derived from their unique expertise and perspectives. Through this collaborative process, the authors ensured that the content of the paper was enriched by the amalgamation of their professional insights and experiences.

### Description of key trends

Key Trend 1. Surge in MAID-PG Requests among Youngsters and Public Platform Given to Testimonials and Narratives presented in the Media.

In general, there is a prevalent perception that the term “MAID” on public platforms is often associated with notions such as ‘dignified dying’, ‘autonomy,’ and ‘self-determination’, reflecting a sense of ‘pure self-efficacy’ in alignment with our highly individualistic society. Consequently, such perceptions are frequently articulated in both general public and clinical settings, where patients often assert their ‘right to MAID’. In reality, patients have the right to request MAID, not necessarily to receive it.

In recent years, Dutch and Belgian media have seldom reported on MAID cases involving terminally ill individuals, despite representing the vast majority of the clinical MAID landscape. Instead, it has now prominently covered the most ‘sensational’ MAID-PG cases. These cases typically involve testimonials from young individuals (under 30 years of age) expressing a persistent desire for or nearing the performance of MAID-PG. Of all the unique stories related to MAID in Dutch and Belgian newspapers and documentaries over the past five years, at least 20 involved testimonials of individuals under the age of 30. However, this number extends beyond mere newspaper coverage. For instance, in Belgium, a 24-year-old patient recently pursued MAID-PG and publicly shared her experience in a newspaper interview, revealing that she created a podcast to destigmatize the topic. Furthermore, this count reflects only unique testimonials, which were often covered by multiple newspapers and media channels. It is crucial to recognize that articles on MAID-PG involving young patients, even those that provide a broader overview rather than detailed individual stories, can still act as triggers for this patient group.

Such media coverage raises concerns regarding the potential influence on vulnerable populations, prompting scrutiny over the possibility of a “contagion effect” or even a “Werther effect,” as observed in the literature on suicidology (19). The “contagion effect” refers to the phenomenon whereby exposure to suicidal behavior within one’s family, peer group, or via media reports can precipitate an increase in suicidal behavior among others. The “Werther effect,” derived from Goethe’s novel *The Sorrows of Young Werther*, characterizes the surge in suicides following the novel’s publication, in which the protagonist’s suicide is central. This term now describes the imitative effect that media portrayals of suicide can exert on susceptible individuals.

Given these established patterns, there is concern that analogous social contagion may extend to MAID-PG, potentially impacting young and vulnerable populations exposed to such narratives. This seems to align with the noticeable increase in younger individuals making MAID-PG requests and highlighted by recent studies among mental healthcare providers (2, 20). More specifically, data from the Dutch Expertise Centre Euthanasia indicated an increase in MAID-PG requests by patients younger than 30 years of age. From 2012 to 2018, there were a total of 206 MAID-PG requests, averaging approximately 29 requests per year (4). This number surged dramatically in recent years, with 781 MAID-PG requests in 2022 and 895 MAID-PG requests in 2023 (8). In Belgium, the number of MAID-PG requests by patients under 30 years of age has also shown a notable increase. Data received from the end-of-life consultation center Vonkel, although not yet publicly available, showed an increase from 4 MAID-PG requests per year during 2015-2016 to 13 requests in 2017. From 2018 to 2021, this average number increased to 20 requests per year. Additionally, from 2019 onwards, there were even reports of minors requesting MAID-PG, although minors cannot be deemed eligible for MAID in Belgium.

In fact, in its 2023 annual report, Expertise Centre Euthanasia explicitly notes that after each instance of media coverage, there is a peak in the number of people requesting MAID-PG (21). Recent qualitative studies raised concerns that the potential for young individuals to imitate self-destructive behavior might not have been fully considered when the law was drafted (22, 23). If the susceptibility to such behavior had been more thoroughly addressed during the drafting process, it could have influenced the development of guidelines for managing MAID-PG requests from younger patients and mitigating potential contagion effects in residential settings. We believe that incorporating these considerations into the legislative process could have helped anticipate and reduce these risks.

It could be suggested, albeit tentatively, that the law may have introduced a new form of death-seeking behavior, aligning with the traditional contagion effect observed in suicidal behavior.

Conversely, there appears to be a notable absence of narratives from patients who have deferred or reconsidered their MAID-PG requests. These counter-narratives, resembling the “Papageno effect” observed in suicide prevention efforts (19), tentatively raise concerns about potential biases in media coverage and its impact on public understanding of MAID-PG. The Papageno effect refers to a protective role, for example, the media can play when discussing suicide in a safe and nonjudgmental manner. It suggests that by not highlighting stories of individuals who have deferred their MAID-PG requests, there could be unintended consequences, such as reinforcing the perception that MAID-PG is the only viable option for those in severe distress and that it represents the ultimate recognition of suffering from (the consequences of) psychiatric illnesses. While providing a platform for young patients to share their experiences with MAID-PG may offer valuable insights, it also presents ethical dilemmas and risks. There is a need to cautiously assess the potential impact on vulnerable individuals, including concerns over feeling pressured to testify or share their stories publicly (2). This pressure may arise from various sources, including efforts to destigmatize MAID-PG or from caregivers involved in the patients’ MAID-PG trajectory, who invite or support these patients to share their stories freely (2, 24). This (often unintended) pressure could inadvertently lead patients to develop a narrower focus on death, potentially overlooking alternative options. This phenomenon can occur because repeated articulation and justification of their decision to pursue MAID-PG may reinforce their commitment to this choice. By constantly explaining their rationale, patients may become increasingly entrenched in their initial decision, thereby solidifying their focus on MAID-PG as the primary solution to their suffering. Additionally, they may grapple with the considerable and difficult-to-anticipate range and potential adverse effects of both positive and negative reactions on their testimonial (24). Furthermore, there may be unintended potential effects of public disclosure on subsequent decision-making related to MAID-PG. The majority of young patients who request MAID-PG revoke their request during the assessment procedure. Publicly announcing one’s wish to die by MAID-PG, e.g. with the intention of breaking the taboo surrounding the topic, may limit their perceived freedom to choose an alternative path. Similarly, patients (re-) engaging in the path of rehabilitation may face challenges when they receive noticeably less public support compared to the support garnered by their pursuit of MAID-PG. This contrast can add further complexity to their journey toward recovery.

Key Trend 2. Mental HealthCare and underlying Societal Aspects.

#### Treatment of MAID-PG trajectories outside regular psychiatric facilities

We witnessed a shift, with an increasing number of patients taking the initiative to seek consultation in specialized end-of-life centers or external professionals with a more permissive approach towards MAID-PG. In addition, there is also a trend observed in Belgium where patients are more frequently directed to these providers by their own treating caregivers, who subsequently delegate the MAID-PG request and procedure to them. In Belgium, for example, approximately 50% of MAID-PG requests were referred by treating clinicians or psychotherapists during 2007-2011 (25). Unpublished data from the end-of-life consultation center Vonkel indicate a significant increase to over 70% in 2018-2022, with referrals by clinicians, psychotherapists, or caregivers. This trend suggests a growing propensity for professionals to delegate MAID-PG requests and procedures to specialized providers.

These specialized end-of-life centers were initially intended as short-term solutions and safety nets for highly complex MAID requests, which are often classified as MAID-PG requests. However recently a cautious trend is visible towards a more *sustainable embedded* practice of these centers managing nearly all aspects of MAID-PG trajectories.

Recent surveys have shown that a minority of psychiatrists are actively engaged in these trajectories beyond referring patients to specialized MAID centers (2, 16). This shift reflects a form of “outsourcing,” where responsibility for assessment and management is delegated to these centers, which now frequently handle the entire MAID-PG procedure, including clarification, formal advice, and the performance of MAID itself.

Ideally, the responsibility for managing MAID-PG procedures should primarily rest with the patient’s treating psychiatrist, supported by the patient’s (multidisciplinary) treating team, possessing an in-depth understanding of the patient’s psychopathology and life context. Specialized MAID centers should be used primarily for additional advice or assistance with the procedure. However, various obstacles complicate and undermine the current landscape of MAID-PG provision. Studies conducted in Belgium and The Netherlands highlight significant deficiencies in knowledge and education regarding MAID-PG of mental healthcare workers (2, 13). In response, new initiatives such as the establishment of Thanet in the Netherlands (see **Box 2**) aim to improve the training of mental health professionals within the general mental health service framework to better handle MAID-PG requests. The outcomes of this initiative are still forthcoming.

Box 2 **The establishment of Reakiro, Oyster Care and Thanet**

**Table:** 

Initiatives in Flanders, Belgium
**REAKIRO** Reakiro is a place in Louvain (2020) and West-Flanders (2022) where individuals considering MAID on psychiatric grounds, and their relatives, can go to. The primary focus of Reakiro is rooted in the rehabilitation approach, characterised by an active orientation towards life, towards (re)discovering meaning, purpose and hope in life, *without* excluding the option of euthanasia. This rehabilitation approach is founded on the following 4 main pillars to qualitative (end-of-life) care: the medical, psychological, social and existential care approach. Within Reakiro, individual support is provided alongside group support in the form of peer-to-peer interactions. https://reakiro.be/ (in Dutch) **OYSTER CARE** In Belgium, the 'Oyster Care' model offers an innovative approach in psychiatry for individuals with Severe and Persistent Mental Illnesses (SPMI), addressing challenges faced by those considering euthanasia when traditional treatments have proven ineffective. by palliative care principles, it prioritizes quality of life through a holistic approach, acknowledging the interconnectedness of various facets of an individual's well-being. 'Oyster Care' endeavors to establish a supportive 'shell' around SPMI individuals, metaphorically representing the external environmental support necessary for the cultivation of a fulfilling life. It acknowledges the reality that complete recovery or reintegration may not always be feasible, and thus, strives to optimize the quality of life attainable within the given circumstances. https://www.crustatievezorg.be/ (in Dutch)

Initiatives in the Netherlands
**THANET** ThaNet was established in 2023 and funded by the Ministry of Health, with the primary objective of augmenting the accessibility and quality of care provided to individuals presenting with a persistent wish to die or a MAID request on psychiatric grounds. Its overarching mission is to systematically facilitate and promote these objectives throughout the entirety of the mental health care system. ThaNet endeavors to enhance the standard of care for individuals expressing a MAID request or enduring a persistent desire for death by instituting a collaborative learning network comprising healthcare professionals operating within mental health care institutions, hospitals, and academic centers. ThaNet facilitates a network where mental healthcare professionals can come together, get acquainted with the subject matter, receive practical guidance and learn how to deal with persistent wishes to die and MAID requests on psychiatric grounds. https://thanet.nl/ (in Dutch)

#### The impact of the MAID-PG trajectory

In addition to the cognitive challenges, such as the complex decision-making required to assess eligibility and the moral dilemmas faced when navigating ethical uncertainties, and the clinical challenges, including managing the intricate and often severe mental health conditions of patients, the emotional impact of participating in MAID-PG trajectories on healthcare professionals demands careful consideration (1, 6, 14).

This strain may arise from the perceived need to balance efforts aimed at suicide prevention with the rigorous assessment required for MAID-PG approval. Furthermore, evidence suggests that pressures from patients’ relatives may intentionally or unintentionally influence psychiatrists’ decisions regarding the (dis)approval or denial of MAID-PG requests (2). Moreover, there is Belgian evidence of relatives engaging a lawyer to exert additional influence on the MAID-PG trajectory, leading to increased unwillingness to (further) engage in MAID-PG trajectory and potentially also increased perceived burden on physicians who are engaging (24, 26). Current literature and guidelines primarily focus on adherence to legal and procedural criteria (1, 2, 27), often overlooking the moral and personal dilemmas faced by psychiatrists themselves, such as ‘moral distress’ due to conflicting values and obligations, as well as ‘demoralization’ characterized by feelings of hopelessness, helplessness, and disillusionment (2, 16).

#### Accessibility and quality of psychiatric care

The observed increase in requests for MAID-PG occurs within a context of strained access to mental health services (14, 15). While this increase cannot be directly attributed to poor-performing mental health care (alone), significant concerns persist regarding poor accessibility and fragmentation of mental health care, particularly for individuals with the most complex and severe mental health needs. Resources are often disproportionately allocated, benefiting those with mild to moderate mental complaints, while individuals facing comorbid disorders or significant societal challenges—such as living alone, low social welfare income, experiencing socio-economic inequalities or public stigma surrounding mental illness—are largely underserved (1).

There has been a significant shift towards providing mental health care in community settings rather than traditional inpatient settings. While this approach emphasizes rehabilitation and resocialization, it may inadequately address the needs of individuals with severe and persistent mental illness (SPMI) who require intensive and specialized care. Furthermore, the emphasis on shorter treatment trajectories may fail to provide sufficient support for long-term recovery and stability.

In Belgium, for example, recent trends show a shift towards more acute and day therapy treatments within the mental health care system. However, this shift is paradoxically accompanied by increased admissions to both public and private psychiatric hospitals. Despite a reduction in the average length of stays—nearly 80% of which are under one month in private psychiatric hospitals—there has been a notable rise in readmissions within the same year and an increase in involuntary admissions. Over the past 20 years, the number of adult psychiatric beds has declined by 6.4%, with chronic care beds decreasing by 39.5% and chronic day and night hospitalization places reducing by 19%. Conversely, there has been a 17.2% increase in acute and specialized beds and a 35.6% rise in acute day and night hospitalization places (14, 28).

Consideration should also be given to factors such as the closure of tertiary care clinics, prompting inquiries regarding the alternative avenues available to patients and thereby underscoring an unaddressed exigency for genuine care. Anecdotal information (not yet published) from Belgium indicates that the closure of one tertiary setting—allegedly for the preventive reallocation of resources—has resulted in many of its patients requesting MAID-PG at a neighboring end-of-life consultation center.

Furthermore, these individuals often experience heightened psychological distress due to various societal burdens and injustices, such as low welfare income and social isolation (2).

The closure of tertiary care clinics raises concerns about the availability of alternative care options, underscoring an unmet need for intensive, comprehensive support. For individuals who may already perceive themselves as burdensome to their families or themselves, these resource constraints can exacerbate their feelings of burdensomeness. This intensified perception may contribute to an increased inclination towards MAID-PG requests. It is important to recognize that these feelings of burdensomeness are often more related to personal or familial contexts rather than broader societal views. Additionally, systemic factors and the evaluation of legal criteria such as ‘medical futility’ and ‘irremediability of suffering’ in clinical MAID practice may further influence (the outcome of) these requests.

#### Navigating complexities in psychiatric care and suicide prevention

The issues discussed extend beyond general medicine and psychiatry, yet they underscore the need for critical reflection within the field of psychiatry, particularly in the context of MAID-PG. Psychiatry must scrutinize its practices and address concerns highlighted by critical psychiatry movements, such as the misuse of power dynamics and the potential for iatrogenic harm. These concerns are particularly pertinent in MAID-PG, where the risk of iatrogenic harm can arise from both the process of requesting MAID-PG and the subsequent interventions (1).

Traumatic experiences reported in psychiatric settings often result from prolonged or involuntary care (1), emphasizing the need for ongoing critical evaluation of psychiatric practices. In the context of MAID-PG, there is an inherent dilemma: balancing the immediate risks of continued suffering against the potential long-term consequences of the intervention. This challenge highlights the necessity for psychiatry to carefully consider how its interventions might impact patients both immediately and in the future.

Engaging in this critical reflection is crucial to ensure that efforts to manage MAID-PG requests do not inadvertently cause additional harm. It is essential that psychiatry remains committed to ethical practices, ensuring that the pursuit of MAID-PG does not exacerbate feelings of burdensomeness and unbearable suffering.

#### Status of palliative care in psychiatry and the call for alternatives

The acceptability of MAID-PG depends not only on the availability of appropriate and accessible standard psychiatric care but also on the provision of quality end-of-life support services. These should underscore a holistic and patient-centered care approach, with a focus on improving quality of life and the relief of suffering, rather than the mere prolongation of life through persistent therapeutic pursuit (18). However, the current state of palliative care in psychiatry remains underdeveloped, with limited resources allocated to address the unique needs of individuals with complex psychiatric issues. Initiatives such as “oyster care” aim to offer specialized palliative psychiatric care for patients for whom traditional therapeutic options are exhausted (29). Nevertheless, these models are underfunded, underdeveloped, and inadequately integrated into mainstream psychiatric practice. Moreover, their focus on patients with severe and persistent mental illness (SPMI) who require long-term residential care leaves a gap in care provision for a proportion of these individuals, who may be too functional for residential care but struggle to navigate the demands of everyday life (the so-called SMI patients, although their condition often can be deemed persistent). This further emphasizes the need for comprehensive, accessible, and sufficiently funded care options.

### Reflection on key trends

#### Main ethical implications

The ethics of MAID-PG require nuanced examination, due to e.g., the complexities inherent in decision-making.

As regards media portrayal, particularly its focus on young patients’ testimonials, requires scrutiny. Exploring the increasing proportion of MAID-PG requests from young individuals could shed light on the plausibility of the Werther-effect hypothesis. While these narratives may offer valuable insights, they risk oversimplifying complex issues and neglecting alternative perspectives. In addition, the lack of balanced representation, especially the absence of narratives from patients reconsidering MAID-PG requests, underscores the need for nuanced media coverage. Empirical evidence on the impact of media portrayal on public attitudes towards MAID-PG would provide compelling support. Future studies examining the correlation between media depictions of MAID-PG and individuals’ perceptions would offer valuable insights into the ethical implications of media representation. Additionally, exploring the role of social media in shaping discourse on MAID-PG, including terminology usage, is crucial. It’s essential to recognize and address the significant disparities and varied expectations among stakeholders involved in psychiatric care, fostering open dialogue, and understanding to develop responsive and inclusive approaches that effectively address diverse needs and perspectives, while also establishing better communication policies for media purposes, akin to those addressing suicide.

Second, the accessibility, adequacy and quality of psychiatric care are crucial. There is a pressing need to enhance regular psychiatric services to provide comprehensive care for underserved individuals, especially those facing significant treatment challenges. In addition, the current challenging psychiatric landscape in both Belgium and the Netherlands, characterized by increasing waiting lists and fragmentation of care, may contribute to heightened perceptions of ‘medical futility’ in psychiatry among patients, their loved ones, and society as a whole. In practice, it is often observed that patients with comorbid disorders struggle to find appropriate treatment. Addressing these challenges may necessitate the implementation of transdiagnostic treatment approaches, which involve addressing barriers to access, and integrating evidence-based practices to ensure equitable and effective care for all individuals requiring psychiatric support.

A more comprehensive and integrated approach to MAID-PG care is also necessary, such that MAID-PG becomes a fully integrated practice within the broader mental health care system. Over the past two decades, awareness and requests for MAID-PG have increased as patients recognize their potential eligibility under legal criteria. This practice is becoming normalized within the patient population, reducing the stigma around requesting it. Addressing MAID-PG in the broader mental health care system is crucial, especially as research indicates that MAID-PG requests often signal a need for additional help rather than a desire to die (16, 30). Integrating its evaluation within therapeutic relationships where offering hope is crucial, is extremely challenging but not impossible. Support can be sought from end-of-life consultation centers. With significant expertise in MAID-PG requests, these centers could provide advisory and supportive help to the patient’s treating psychiatrists and, by extension, the entire treating team, aiding in assessment procedures without taking over all the responsibilities.

Moreover, integrating recovery-oriented and palliative approaches in psychiatry is urgent, especially where MAID legislation includes psychiatric conditions. MAID-PG requests should stem from unalleviated suffering *despite* comprehensive and adequate care, not due to incomprehensive and inadequate care. Finally, guidelines and research often focus on psychiatrists, neglecting the roles of other mental health professionals like nurses, psychologists, moral consultants, and social workers (23). Their roles and support needs must be addressed within an integrated approach to MAID-PG care.

Examining the ethical implications of all these challenges through the lens of patient - as well as health caregiver- autonomy, beneficence, non-maleficence, and justice would provide a deeper understanding of the complexities involved.

Third, exploring alternative models of end-of-life care could offer insights into addressing (some of) these ethical concerns. Exploring the development of palliative psychiatry approaches for cases where resocialization and recovery prospects are limited holds great promise. Drawing parallels to the supportive measures seen in somatic medicine during the inception of MAID laws can provide valuable insights and guidance in this endeavor. Palliative psychiatry approaches should focus on alleviating suffering, enhancing quality of life, and providing holistic support to individuals with severe and persistent mental illness who may be considering end-of-life options. However, as we embark on this exploration, it is essential to prompt societal reflection on the reality of incurable psychiatric conditions. We must critically evaluate our expectations of psychiatric care and acknowledge its limitations, recognizing that the treatment potential for some patients may be overestimated. This calls for a balanced perspective that acknowledges both the possibilities and limitations of psychiatric treatment outcomes. It is crucial to ensure that individuals facing end-of-life decisions receive compassionate and comprehensive care that respects their autonomy and dignity while also acknowledging the challenges and limitations inherent in psychiatric care.

Fourth and from a broader clinical perspective, especially at earlier stages in the mental healthcare continuum, it is crucial to address prompted or expressed death wishes within every clinical encounter. This entails creating a calm environment conducive to exploring the underlying motives for these thoughts. This approach aims to prevent the escalation of suppressed thoughts into crises that are difficult to manage, potentially leading to suicidal behavior or requests for MAID-PG. Furthermore, there is a pressing need to enhance professionals’ understanding and proficiency in navigating the delicate balance between taking suicidality seriously without setting off alarms and introducing hope without disregarding the suffering involved in such discussions. Additionally, we must gain more insight into the relationship between fluctuations over time in suicides and MAID-PG in order to comprehend the overlapping mechanisms.

#### Societal implications and their impact on clinical practice, and vice versa

It’s essential to recognize the nuanced relationship between MAID legislation and broader societal dynamics, avoiding oversimplified causal interpretations. While MAID laws may have an impact, it’s crucial to consider the influence of various societal factors on individuals’ decisions regarding MAID-PG. A comprehensive understanding of the societal landscape is necessary for informed discourse and shared decision-making in clinical practice.

Addressing broader societal factors contributing to individuals’ distress and desire for MAID-PG requires a multifaceted approach. This entails not only improving mental health services but also challenging societal attitudes towards mental illness and end-of-life care. Research demonstrating the impact of discrimination, socioeconomic disparities, stigma, and systemic barriers on individuals’ mental wellbeing, their access to mental health care and their decision-making regarding MAID-PG is to be recommended. Moreover, it is important to delve deeper into the many other societal factors contributing to the distress of youngsters, considering that observed increases in MAID-PG requests coincide with rising rates of indirect self-destructive behaviors, such as self-mutilation, food restriction and suicide (31). One aspect that may need closer examination is the potential role of co-rumination (32). Co-rumination involves excessively discussing and dwelling on problems or negative emotions with other people without making progress towards resolution. This pattern may involve repeatedly discussing the same concerns, seeking validation, and venting emotions, which can perpetuate and worsen mental distress. In the context of MAID-PG trajectories, individuals may engage in prolonged discussions or rumination about their decision with peers and online platforms, potentially reinforcing negative emotions and narrowing focus on death. Furthermore, this can complicate decision-making and intensify (internalized) pressure on individuals considering MAID-PG. Practitioners should be aware of co-rumination’s impact and guide conversations towards constructive problem-solving and coping strategies, while also addressing broader social and psychological factors contributing to this pattern. Policies and interventions should ensure that individuals facing end-of-life decisions have access to comprehensive support networks and resources to facilitate informed decision-making and well-being. This could include addressing one-sided media attention, as discussed above, to provide a more balanced portrayal of end-of-life options and support individuals in making decisions aligned with their values and preferences. Such an approach can mitigate the negative effects of co-rumination and empower individuals to make choices aligned with their values.

## Conclusion

Fostering a culture of ongoing reflection and learning is paramount to be better positioned to address the multifaceted challenges in psychiatric care and end-of-life decision-making. To address the complexities of MAID-PG effectively, collaboration among various stakeholders—research institutions, policymakers, review committees, media, and psychiatric associations—is crucial. Research institutions can advance knowledge through independent and rigorous studies, policymakers can develop and refine regulations, review committees can ensure both transparency and compliance with legal and ethical standards, the media can shape public discourse responsibly, and psychiatric associations can provide guidelines and training. By integrating these diverse perspectives and maintaining a commitment to ethical practice, principles of autonomy and compassionate care for individuals facing complex end-of-life decisions can be upheld more effectively.

Amid concerns and critiques, it’s important to highlight existing initiatives already addressing some of the societal challenges associated with MAID-PG (see **Box 2** for more information on and references to these initiatives). Initiatives like ThaNet in the Netherlands and REAKIRO in Flanders demonstrate proactive steps toward improving knowledge on and care for individuals with MAID-PG requests or persistent death ideation within the mental healthcare system. Furthermore, while innovative models like ‘Oyster Care’ in Belgium underscore the significance of holistic approaches in psychiatry, there is a need to expand and strengthen initiatives for greater overall benefit. The broader reflections offered in this paper aim to encourage more nuanced and informed discussions in the current public discourse.

## Data Availability

The original contributions presented in the study are included in the article/supplementary material. Further inquiries can be directed to the corresponding author.
